# Supplementary material for: Functionally important residues from graph analysis of coevolved dynamic couplings
Source: eLife. 2025 Mar 28;14:RP105005. doi: 10.7554/eLife.105005 (PMC11952748; doi:10.7554/eLife.105005)
Supplement: Supplementary file 1. — (a) Hydrophobic nodes in SHV-1. (b) Dynamic descriptors and number of residue pairs detected. (c) Number of residues in each community detected using only the coevolution scores. (d) Summary of molecular dynamics simulation systems. [file elife-105005-supp1.docx]

**Supplementary Tables**

**Functionally Important Residues from Graph Analysis of Coevolved Dynamical Couplings**

Manming Xu, Sarath Chandra Dantu, James A Garnett, Robert A Bonomo, Alessandro Pandini*, and Shozeb Haider*

***Corresponding author:**

Shozeb Haider

Email: [Shozeb.haider@ucl.ac.uk](mailto:Shozeb.haider@ucl.ac.uk)

ORCID: 0000-0003-2650-2925

Alessandro Pandini

Email: [alessandro.pandini@brunel.ac.uk](mailto:alessandro.pandini@brunel.ac.uk)

ORCID: 0000-0002-4158-233X

**This PDF file includes:**

Supplementary Tables

**Supplementary file 1a** Hydrophobic nodes in SHV-1 (1)

| **Nodes** | **Residues** | **Residue No.** |
| --- | --- | --- |
| α2 | VVLCGAVLA | 74-82 |
| α3 | DLV | 101-103 |
| α4 | SPV | 106-108 |
| α5 | AAAI | 124-127 |
| α6 | SAA | 133-135 |
| α6 | LLL | 137-139 |
| α9 | SMA | 185-187 |
| α10 | RLSA | 198-201 |
| α11 | SVL | 223-225 |
| β7 | FIA | 230-232 |
| β8 | ALL | 248-250 |
| β9 | AVV | 260-262 |
| α12 | AGIG | 280-283 |

**Supplementary file 1b** Dynamic descriptors and number of residue pairs detected

| **Dynamic Descriptors** | **Number of Pairs Detected** | |
| --- | --- | --- |
|  | **SHV-1** | **PDC-3** |
| R_g_ | 0 | 0 |
| PC1 | 571 | 185 |
| PC1_partial | 161 | 211 |
| TC1 | 119 | 172 |
| TC1_partial | 339 | 216 |
| gRMSD | 26 | 0 |
| pRMSD | 203 | 117 |
| dRMSD | 0 | 0 |
| gSASA | 23 | 0 |
| pSASA | 18 | 62 |
| volume | 0 | 0 |
| hbond | 0 | 0 |

**Supplementary file 1c** Number of residues in each community detected using only the coevolution scores

|  | **Community 1** | **Community 2** | **Community 3** | **Community 4** |
| --- | --- | --- | --- | --- |
| SHV-1 | 84 | 50 | 44 | 86 |
| PDC-3 | 93 | 57 | 82 | 126 |

**Supplementary file 1d** Summary of molecular dynamics simulation systems

| **System** | **Number**  **of Trajectories** | **Frames for**  **Each Trajectory** | **Total Simulation Time (μs)** | **Stride** | **Total Frames**  **After Stride** |
| --- | --- | --- | --- | --- | --- |
| SHV-1 | 593 | 600 | 35.58 | 10 | 35580 |
| PDC-3 | 100 | 3000 | 30 | 10 | 30000 |

*All trajectories were simulated with a time step of 0.1 ns

**References**

1. E. Olehnovics *et al.*, The Role of Hydrophobic Nodes in the Dynamics of Class A beta-Lactamases. *Front Microbiol* **12**, 720991 (2021).
